# The impact of direct provision accommodation for asylum seekers on organisation and delivery of local primary care and social care services: A case study

**DOI:** 10.1186/1471-2296-12-32

**Published:** 2011-05-15

**Authors:** Hans-Olaf Pieper, Pauline Clerkin, Anne MacFarlane

**Affiliations:** 1 Discipline of General Practice, National University of Ireland, Galway, Galway, Ireland

## Abstract

**Background:**

Many western countries have policies of dispersal and direct provision accommodation (state-funded accommodation in an institutional centre) for asylum seekers. Most research focuses on its effect on the asylum seeking population. Little is known about the impact of direct provision accommodation on organisation and delivery of local primary care and social care services in the community. The aim of this research is to explore this issue.

**Methods:**

In 2005 a direct provision accommodation centre was opened in a rural area in Ireland. A retrospective qualitative case study was designed comprising in-depth interviews with 37 relevant stakeholders. Thematic analysis following the principles of framework analysis was applied.

**Results:**

There was lack of advance notification to primary care and social care professionals and the community about the new accommodation centre. This caused anxiety and stress among relevant stakeholders. There was insufficient time to plan and prepare appropriate primary care and social care for the residents, causing a significant strain on service delivery. There was lack of clarity about how primary care and social care needs of the incoming residents were to be addressed. Interdisciplinary support systems developed informally between healthcare professionals. This ensured that residents of the accommodation centre were appropriately cared for.

**Conclusions:**

Direct provision accommodation impacts on the organisation and delivery of local primary care and social care services. There needs to be sufficient advance notification and inter-agency, inter-professional dialogue to manage this. Primary care and social care professionals working with asylum seekers should have access to training to enhance their skills for working in cross-cultural consultations.

## Background

Globalisation and movement of people from their countries of birth are occurring more rapidly than ever before. Migration is often a result of forcible displacement because of complex social, political or environmental events. Worldwide, at the end of 2009, a total of 43 million people had been forcibly displaced, the highest number since the mid-1990s. Of these, 983,000 people sought asylum in other countries [1]. The right to seek asylum is stated in the Universal Declaration of Human Rights, which states that 'everyone has the right to seek and to enjoy in other countries asylum from persecution' [2]. An asylum seeker obtains asylum if he/she meets the United Nations' definition of a refugee: someone who '*owing to a well-founded fear of being persecuted for reasons of race, religion, nationality, membership of a particular social group, or political opinion, is outside the country of their nationality, and is unable to or, owing to such fear, is unwilling to avail him/herself of the protection of that country' *[3]. In 2009 the total number of refugees was 15.2 million. Europe hosted 16% of the world's refugees, with 358,000 new asylum claims during 2009 [1]. These global migration patterns have relevance for Ireland in that they are contributing to recent and unprecedented patterns of inward immigration [4].

It is important that host healthcare systems respond appropriately to asylum seekers' healthcare needs for two main reasons. First, they are a group with complex health and social care needs which are influenced by a myriad of factors including the experiences that led to their need for asylum (e.g. persecution and violence), the experience of being an asylum seeker in an unfamiliar country (e.g. language barriers, lack of knowledge of available services and supports, hostile responses from host communities) and the challenges inherent in that process of seeking asylum in their host country (e.g. long delays in the application process) [5,6]. Second, asylum seekers have the right to health care. According to the Universal Declaration of Human Rights, equal access to health care is an acknowledged human right [2]. Also the World Health Organization (WHO) Alma Ata Declaration 1978 states universal access to health care as its goal [7]. However, health policies towards asylum seekers differ significantly between the EU countries, leading to debates about whether health needs of asylum seekers are being adequately met or not [8].

Many western countries, including Ireland, have policies of direct provision accommodation. This is a specific accommodation policy for asylum seekers whereby they are accommodated in full-board accommodation centres run in an institutional style rather than private or self-catering accommodation. Typically, direct provision centres are spread across the country as part of a related 'dispersal policy'. This involves moving asylum seekers to accommodation centres in different regions, the idea being to share the resource burden more equally among a wide range of local authorities [9].

National and international reports argue that direct provision accommodation is a violation of basic human rights [10,11] and research indicates that it is linked with poverty, and poor physical and mental health [6,12-14]. Studies have focused on the issue of provision of health care to asylum seekers [12,15-17]. However, we know very little about the specific issue of the impact of dispersal and direct provision on the organisation and delivery of local primary care and social care services. For instance, arguably, there is an impact on the work of general practitioners and public health nurses because the volume and nature of their work would be affected by the arrival of asylum seekers in their local setting. Of course this, in turn, could impact on asylum seekers' access to, and experiences of, primary care and social care consultations.

An important study in the UK has examined this issue in relation to health care. It showed that healthcare providers were concerned that decision making about placement of asylum seekers seemed to be based purely on accommodation availability rather than the capacity of local healthcare services. Healthcare providers in this study perceived that there was inadequate co-ordination by the National Asylum Support Service (NASS) and that this was the main barrier to effective and efficient delivery of health care for asylum seekers. Some specific issues mentioned were lack of notification about incoming dispersed asylum seekers, departure from agreed language clusters, inconsistent standards of NASS accommodation providers, high mobility of asylum seekers within the dispersal system, and problematic bureaucracy intended to support asylum seekers. Most healthcare providers said that their principal problems arose from the number, diversity and irregular flow of asylum seekers [18].

Another important study focused on the Community Welfare Service in Ireland and examined the experiences of statutory service personnel at the frontline in responding to basic needs of asylum seekers and refugees. This study highlighted the challenges for staff when working with asylum seekers. They had difficulties working with, and understanding, cultural differences. They also had difficulties with language differences and communication. They desired intercultural training to advance their knowledge and skills. They also described problems with inter-agency communication and collaboration which negatively affected their frontline role [19].

However, these studies are relatively old and there has been no recent analysis of this issue in relation to general practice and other primary care and social care services. The aim of this research is to explore, with all relevant stakeholders, the impact of direct provision accommodation for asylum seekers on the organisation and delivery of the local primary care and social care services, and to identify recommendations for future policy and practice. In the Irish context, asylum seekers are people who have applied for asylum and are awaiting a decision. Further details of the study context are provided below.

### Context - the Republic of Ireland

**Figure 1:**
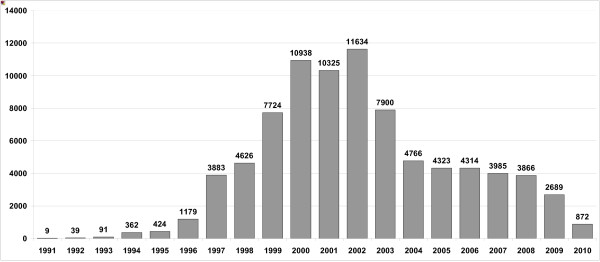
**Asylum applications in the Republic of Ireland, 1991 to May 2010 **[21].

The arrival of migrants, refugees and asylum seekers in Ireland and the ensuing diversity is part of an unprecedented pattern of inward migration into Ireland. Currently one out of ten persons living in Ireland comes from a non-Irish background [20]. The number of asylum seekers in Ireland increased dramatically from only nine in 1991 to a peak of 11,634 in 2002, before falling off in 2003 and down to 2,689 in 2009 (see Figure 1[21]). Various domestic policies are likely to have contributed to the downward trend in applications since 2003 [22]. The decrease was also part of a larger drop in asylum applications lodged in industrialised countries between 2002 and 2006, reported by the United Nations High Commissioner for Refugees [23].

The Reception and Integration Agency (RIA) is a section of the Department of Justice, Equality and Law Reform and is responsible for coordinating the provision of services to both asylum seekers and refugees, coordinating the implementation of integration policy for all refugees and persons granted leave to remain in the state, and responding to crisis situations which result in large numbers of refugees arriving in Ireland within a short period of time [24]. There is a dispersal policy in place which means that asylum seekers are living in a range of urban and rural areas around the country. Typically, accommodation centres are old hotels or hostels and they operate on the basis of a commercial arrangement between the state and the accommodation centre owner. Residents receive €19.10 per adult and €9.60 per child per week [25]. They often live in shared, crowded rooms with very basic facilities and amenities. There is evidence about the negative impact which direct provision accommodation has on asylum seekers' mental health [13].

The Health Service Executive (HSE) is responsible for providing health and personal social services for everyone living in the Republic of Ireland. The HSE operates a two tier system whereby people with lower incomes are eligible for a General Medical Scheme (GMS) and are provided with a medical card which entitles them to free medical care. All asylum seekers have this entitlement. The HSE nominates liaison officers who are contact persons for the Immigration Officer.

## Methods

### Research design

This is a qualitative study using a retrospective, case study design [26]. A case is a bounded system explored over time. In this study, the case is a direct provision accommodation centre located in a rural area in Ireland, referred to here as Accommodation Centre A. The nearest village is located 4 km away. There is no access to public transport from the accommodation centre.

The case study was initially designed to explore the opening of the accommodation centre, which happened with minimal advance notice for key stakeholders. During the course of the research, the accommodation centre was closed, also with minimal notice to stakeholders. The case study was thus extended to capture stakeholders' experiences around the closure of the centre as well.

### Ethical approval

Ethical approval for the study was obtained from the Research Ethics Committee of the National University of Ireland, Galway.

### Sampling and recruitment

Purposeful sampling strategies [27] were used to identify 'information rich' participants. Three stakeholder groups were identified for this case study:

• Frontline staff (statutory and non-statutory)

• Service users (local service users and asylum seekers)

• Policy makers and service planners (national and regional).

Age and gender were also included as sampling parameters to ensure that a wide range of views were documented.

Potential participants were invited to take part in the research by letter or personal contact. Responses were monitored to create a database of interested participants from which sampling occurred. The majority of people who were approached did participate in the study. There were some people who did not take part. One frontline staff, a receptionist of a GP surgery, was not comfortable participating in a research project. Two local service users felt that they were unable to contribute enough to the research question to merit participating. Three asylum seekers did not take part because they felt uneasy about signing a consent form and having their interview tape recorded. The final database comprised 37 interested participants, all of whom were interviewed (see Table 1).

**Table 1:** Overview of research participants (N = 37)

Frontline staff (n = 16)	
**Statutory**	**Non-Statutory**

• General Practitioners (n = 4)	Non-Governmental Organisations
• Area Medical Officer (n = 1)	• Refugee Support Group (n = 1)
• Public Health Nurses (n = 2)	• Family Resource Centre (n = 1)
• Directors of Public Health Nursing (n = 2)	Direct Provision Accommodation centre
• Health Promotion Officer (n = 1)	• Managers (n = 2)
• Social Worker (n = 1)	
• Community Welfare Officer (n = 1)	

**Service users (n = 17)**	

**Host Community**	**Migrant Community**
• Local service users (n = 7)	• Asylum seekers (n = 10)

**Policy makers and service planners (n = 4)**	

**Regional**	**National**
Health Service Executive	Reception and Integration Agency (RIA)
• Development Officer, Primary Care Department (n = 1)	• Staff (n = 2)
• Liaison Officer (n = 1)	

### Data collection and analysis

Case studies use extensive and multiple sources of information in data collection to provide a detailed in-depth picture of the case. In this study, in-depth interviews [28], relevant documentation such as related articles in the local newspapers, and reflective retrospective observations by researcher Hans-Olaf Pieper (HOPieper), who was also a GP involved in the case, were drawn on for analysis.

In the fieldwork with participants, semi-structured in-depth interviews were carried out by HOPieper and social scientist Pauline Clerkin (PClerkin). Participants were asked to complete a consent form at the time of data collection. Interviews were recorded with participants' permission with a portable device and later fully transcribed. In keeping with the iterative nature of qualitative research, data analysis occurred immediately as the data collection began and was continued throughout the process of investigation. The understanding gained during early data analysis shaped decisions about subsequent phases of data collection, e.g. it led to 'snowball' samples [27]. A thematic analysis by all three authors following Silverman [29] and Morse [30] was conducted using NVivo software [31]. Fieldwork using in-depth interviews continued until theoretical saturation occurred, that is, that no new themes were emerging in the analysis.

## Results

**Figure 2:**
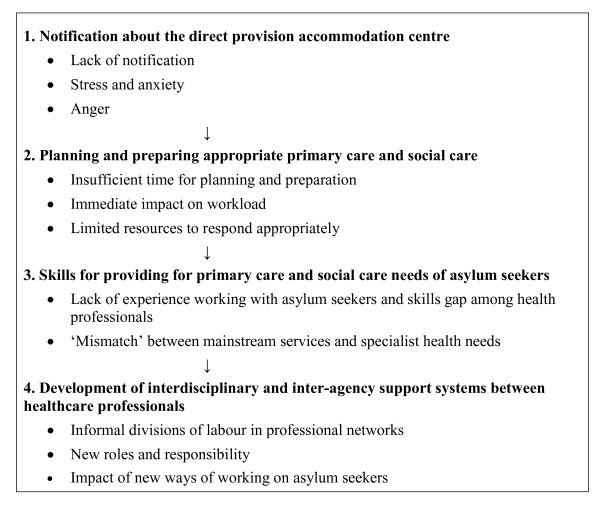
**Overview of themes**.

There were four main themes identified in this analysis and these are shown in Figure 2 along with the sub-themes relevant to each one and the way in which the themes are interrelated. The first theme 'notification about the direct provision accommodation centre' has a 'knock on' effect on the second, 'planning and preparing appropriate primary care and social care' in that there was a lack of notification about the opening of the accommodation centre which meant that there was insufficient time to plan and prepare appropriate health and social care services. There was also no time to address the skills gap among health professionals to meet the needs of the asylum seekers, which is the third theme 'skills for providing for primary care and social care needs of asylum seekers'. Finally, the fourth theme relates to a key outcome on the ground, 'development of interdisciplinary and inter-agency support systems between healthcare professionals'.

### 1. Notification about the direct provision accommodation centre

It was known at the outset of this study that there was poor notification about the opening of the direct provision accommodation centre, and so it is not surprising that it features as a main theme in the analysis. This theme offers a systematic account and analysis of the effects of the poor notification.

#### Frontline staff

##### Statutory services

Some primary care and social care providers got very short notice. Others got no notice at all and heard the news locally, either on the day that the asylum seekers arrived or after they had arrived. This was the case across all statutory services.

"I personally remember being off on the Friday and getting the phone calls to say this was happening and did I know, em... there was no prior consultation, there was no, there was no preparatory time really, em... and that was very flooring" (GP 1).

"I just got a phone call from Dublin and all those birth times, children were coming through the fax so it was kind of a big shock for me. I just didn't know what was happening and my assistant director, when I rang her she wasn't aware of anything, you know so it was kind of gruesome really you know" (Public Health Nurse 1).

When Accommodation Centre A was closed, again there was lack of notification. Health and social services were only informed about the closure when word spread informally.

##### Non-statutory services

The Refugee Support Group got no prior notification but got a phone call asking if they would help support the asylum seekers on the same day they arrived.

#### Service users

##### Local service users

The lack of notification about the opening of Accommodation Centre A was also a cause of stress and anxiety for the local community at two levels: as local residents in the community, and also as service users of the local health and social care services. There were different aspects to the community's response. In keeping with newspaper reports at the time, there was quite a lot of negativity. The lack of notification about the opening of the accommodation centre made community members feel that they had been "left out of the loop" of the decision making processes, and some had the feeling of having been deceived. For one participant there was negativity toward asylum seekers themselves, for instance concerns over issues such as disease and a potential increase in crime rates. Service user participants explained that, over time, these fears were not borne out. Others discussed their concerns in relation to practical issues that had implications for the asylum seekers and the wider community. For instance, some emphasised the fact that the location of the accommodation centre was unsuitable because it was a rural location with poor transport links. There were concerns that this would cause isolation among the asylum seekers. Another practical concern was that of overload on the sewerage system, given that there had been problems in that area previously.

##### Asylum seekers

The sudden opening was not considered problematic for the interviewed asylum seekers. Interestingly, asylum seekers expected some antagonism from local service users about their arrival, but they were surprised that they did not experience negativity.

"We were surprised, we thought that people in the village they didn't like us, that we should all leave, that they should close the hotel, but we never seen or come across any villager, at the end they were nice to us. The few we met were really nice. They were even bringing things to the hotel for us" (Asylum Seeker 5).

#### Policy and service planners

Interviews with staff from the RIA and the HSE reveal some insight into the lack of notification about the opening of the accommodation centre. Both acknowledged difficulties as a result of it and explained and emphasised the unusual circumstances.

First, an amendment to the Irish constitution in 2004 meant that from January 2005 it was no longer possible for persons born in Ireland to obtain automatic Irish citizenship. Via a special scheme the Department of Justice, Equality and Law Reform accepted applications to remain in the state under the previous legislation up to March 2005, on the basis of such parentage [32]. As a result, in addition to the regular inward flow of asylum seekers for that time (4,323 in 2005), 17,917 applications were submitted under this scheme [33]. The upshot was that the RIA had to provide around 8,000 additional people with accommodation (personal communication, RIA), which put huge pressure on the agency. The RIA explained that a search for suitable locations had to take place and that selection decisions about suitable accommodation centres had to be made quickly. Consultations with the community are generally not done, because in previous cases public debate and opposition has led to scenarios in which accommodation centres could not be opened. In particular the urgency of the situation was seen as over-riding any need for consultation with the community. In addition, consultation with the HSE did not take place as it was feared discussion would be passed on to the local community and hinder the opening of the accommodation centre.

Second, at the time the HSE liaison officer with a remit for asylum seekers was on sick leave. Therefore, there was no point of contact for the RIA at the local HSE. This meant that there was a significant 'gap' in communication that inhibited planning and preparation for this new development.

### 2. Planning and preparing appropriate primary care and social care

The lack of time for planning and preparing appropriate health and social care had an impact on all frontline staff. There was a range of specific problems and challenges. These are shown in Table 2 below. It is clear that some were shared by staff (e.g. lack of access to medical records, overcrowding in health and social care facilities) and others were specific to certain staff (e.g. increased need for foster care for social work services).

**Table 2:** Summary of perceptions of frontline staff

Statutory	
General Practitioners	• Most asylum seekers young mothers, many children, some pregnant within days of delivery
	• No medical cards available
	• No medical notes available
	• No clarity about previous tests - possibly repeated unnecessarily
	• Waiting room overcrowded
Public Health Nurses	• No medical notes available
	• No clarity about previous procedures, e.g. vaccines
	• Waiting room overcrowded
Community Welfare Officer	• Waiting room overcrowded
	• Local service users' ill-feeling towards asylum seekers
	• Local service users stopped attending
Social Worker	• Foster places for children during hospitalisation of asylum seekers required, drain on resources

**Non-statutory**	

Refugee Support Group	• Out of their geographical region and official remit
	• Limited resources to respond

#### Frontline staff

##### Statutory services

From the community-based frontline staff point of view, the lack of notification meant that there was insufficient time to plan and prepare appropriate primary care and social care services. This caused stress at a professional level. They also experienced an immediate impact on workload and had limited resources to deal with this. This was felt most by the GPs. Most asylum seekers were young mothers with small children. Some were pregnant within days of delivery with immediate health needs. The most dramatic example relates to an asylum seeker who went into labour just 48 hours after arrival at the accommodation centre. She was brought to the hospital with no ante-natal history available and staff had a lot of difficulties. In light of the urgency the GPs initially provided their service free of charge, as the vast majority of the asylum seekers had recently arrived in Ireland, and did not yet have a medical card. Before obtaining a medical card it was necessary to clarify which of the three local GPs would agree to accept the asylum seekers as patients. This decision-making process was time consuming and required several meetings of the GPs. Some of the GPs felt uneasy accepting more patients, as they already had very busy practices.

Having to make such long-term decisions under pressure caused anger and anxiety among GPs:

"So there was a lot of frenzied activity and a lot of concern and it was very stressful" (GP 1).

"I suppose I had a busy practice, em... I wasn't in any way ready to take on even, you know, 100 new medical card patients" (GP 1).

General practice waiting rooms were overcrowded due to patients arriving in great numbers and being unable to leave because of lack of transport to and from the accommodation centre. The concerns of the GPs about this were also recognised and acknowledged by the Development Officer of the Primary Care Department in the HSE.

In the course of the meetings and negotiations between the GPs, it emerged that one GP was in a position to accept all asylum seekers as patients. Following this decision, medical cards were provided very quickly, which was positive given that it normally takes a couple of weeks for applications to be processed.

Anxieties and concerns were not restricted to the GPs, but permeated throughout the frontline statutory and non-statutory services. For example, it was generally felt that the lack of notification had impacted on workload, whereby prior notice would have meant that preparations could have been made in advance. For instance, there was a particular problem arising from the fact that no records were available for the asylum seekers about prior medical history, treatments or procedures. Very few of the asylum seekers themselves had been given notes to bring with them. Advance notice of their arrival may have given some time for notes to be located. In the absence of notes, there was duplication of tests and procedures which resulted in increased costs for the health services.

When the accommodation centre closed (again without sufficient notification to service providers) there was regret across primary care and social care services mainly because continuity of care was, again, interrupted. This was particularly the case for the GP and the public health nurse. Interestingly and as a contrast, the community welfare officer explained that when she got informal advance notice about the closure of the accommodation centre she was able to prepare files for colleagues in appropriate office on the receiving end. This minimised discontinuity of care which she was very pleased about.

A specific problem for social work staff related to childcare, which was a major issue for the asylum seekers because they did not always have someone to care for their children while they were at appointments or in-hospital stays. As a result the social worker often had to organise foster places, which she perceived to be traumatic for mothers and children, and a significant use of health service resources.

##### Non-statutory services

The Refugee Support Group also had very short notice of the opening of Accommodation Centre A. While the geographical area was out of their remit and they were under-resourced, they felt that their support was needed.

### 3. Skills for providing for primary care and social care needs of the asylum seekers

#### Frontline staff

Most statutory frontline staff emphasised that they did not have experience working with asylum seekers. They found it difficult to suddenly respond to their needs because they felt they lacked skills to do so.

"Well it was very difficult ...to suddenly be faced with the situation where you were going to have a whole population group with probably very different needs to what you were used to... you didn't have any direct experience in that area... there was no kind of preparatory work" (GP 1).

GP3 explained that he lacked skills to communicate with patients with limited English proficiency, work with interpreters, recognise cultural diversity such as differences between one's own and patients' health beliefs, manage patients' expectations about health service provision, handle clinical issues such as uncommon dermatological diseases on coloured [sic] skin, and provide medico-legal reports for survivors of torture.

A public health nurse participant discussed a particular 'gap' in experience and skills in relation to mental health needs of asylum seekers (an issue raised by several service providers).

"Well I'm not a psychiatric nurse, and most of the clients have had either post-traumatic stress, or depression, or anxiety, or suicidal tendencies, they're post-trauma, post-rape, post-torture, and I mean, like I dealt with it, but I was only giving a band aid service as well, because I'm not qualified like that, you know" (Public Health Nurse 3).

This lack of experience and gap in experience and skills in relation to mental health is indicative of a more general 'mismatch' between the services designed for 'mainstream' service users and asylum seeker service users living in the direct provision centre under study. In the case of the health promotion officer and public health nurse, the work that they were planning to do with the asylum seekers based on work in 'mainstream' services was just not what the asylum seekers needed, or indeed wanted. The health promotion worker found that the methods and sessions she was used to working on with other groups did not work with this group, and she felt out of her depth in dealing with issues in which she didn't feel qualified. However, over time and with increased discussion with the asylum seekers they came to understand each other and the issues that were of most concern.

#### Service users - asylum seekers

The asylum seekers found it difficult at first to adjust to a different primary care and social care system and it took a while for them to understand how the system works, particularly in terms of medical treatment. This resonates with views of the community welfare officer, who found that the lack of information that the asylum seekers were given about supports and services led to extra pressure on some services. Newly arrived asylum seekers would sometimes approach a service which was not designed for their presenting health or social care needs, which is unsatisfactory for the service user and provider alike.

### 4. Development of interdisciplinary and inter-agency support systems between healthcare professionals

There was shared concern among frontline staff that solutions must be sought and a number of formal meetings were organised to attempt to address some of the main concerns. For instance, the RIA organised a formal meeting three weeks after the opening of the accommodation centre, which all relevant frontline staff were invited to attend. However, despite this *formal *meeting, frontline staff from statutory services felt that there was no formal response, that is no concrete actions or supports for their day-to-day work. What transpired was that many health and social service professionals had to proactively and *informally *seek support from each other to help them with their new work. As a result of these informal meetings, divisions of labour in professional networks were agreed. For example, one GP agreed to take on responsibility for the entire group of asylum seekers. This was because he had a smaller practice size than the other GPs in the area, and was therefore able to provide a solution to the potential workload issues. He also had an interest in migrant health.

Also, frontline service providers negotiated new roles and responsibilities for themselves individually or in collaboration with other colleagues. The dedicated GP engaged in collaboration with a local non-governmental organisation (NGO) to promote integration of asylum seekers with the local community.

"So I made contact with (Refugee Support Group). I didn't even know that that exists, so, and I got information leaflets... I tried to educate the people about the asylum seekers, and anti-racism strategies, put them out in the waiting room, and tried, in a way, to make the presence more welcome, basically to bring people together" (GP 3).

Also, a chance encounter between the health promotion worker and the public health nurse in the area led to a shared sense of the problem of providing mainstream services to asylum seekers who have specialist needs. As a result they organised a new and appropriate work programme of addressing the needs of the group. For instance, they identified the relevance of group work rather than one-to-one work with the asylum seekers and modified their work accordingly.

"I had the expertise of the group work, so for instance I could manage the group, and instead of her (Public Health Nurse) doing one-to-one sessions only, which she had been doing her best to do before, and they weren't attending, or very few of them were attending, they could also start to become a group, and this was very powerful." (Health Promotion Officer)

#### Impact of new ways of working on asylum seekers

The existence of a dedicated GP for the asylum seeking service users had a positive impact on their experience and perception of the health services, in terms of the speed of accessing a GP and of the service provided to them. The asylum seekers who were interviewed did not feel that there were any problems relating to overcrowded waiting rooms, nor did they feel like they had experienced any negative attitudes from other patients.

"Mm, well I think GP-wise we didn't have any problems, if my children are really sick, I didn't have any problem with the GP" (Asylum Seeker 4).

## Discussion

The present research focused on the organisational impact of direct provision accommodation on local primary care and social care services. It was designed to examine the experience of all stakeholders involved in order to make recommendations for policy and practice.

### Summary of key findings

A major finding is that the lack of notification about the opening and closure of Accommodation Centre A was very problematic for frontline service providers. This finding is not surprising, resonates with previous research [18,19] and also underlines our interest in conducting this case study in the first place. What is important about this finding is that it provides in-depth understanding about the nature of problems experienced at the time by different stakeholder groups. It also highlights the need for increased education/training in clinical knowledge and skills for service providers involved in the care of asylum seekers. A major finding is the *psycho-social impact *of the situation on frontline service providers. They were stressed about the pressurised situation in which they found themselves and were angered by the strain they experienced.

Health professionals were stressed by the *clinical challenges and problems *that arose because health records were unavailable, the scope for informational continuity of care [34,35] was nil, and there was duplication of clinical tests and therefore unnecessary use of healthcare resources. We know that continuity of care is problematic across institutional and professional boundaries [36] and that it is important that information 'follows the patient' to all healthcare contexts where they seek care [37]. Lack of notification about dispersal and opening and closing of direct provision accommodation centres makes continuity of care highly demanding and often impossible. This is very problematic because asylum seekers, like other vulnerable patient groups who have complex case histories [38-40], benefit from good continuity of care. To enhance informational continuity [34], at least, frontline service providers need more notification of the openings and closings of accommodation centres. This would, for example, provide frontline staff with time to examine the available resources, access appropriate medical records, and assess the potential utility of mainstream services for the new service users versus the need for more specialised services.

Some stress for frontline service providers related to their interactions with asylum seekers in cross-cultural consultations. Frontline staff in statutory services reported that they did not feel they had the necessary *skills *to work to a high quality level with asylum seekers and their specialised healthcare needs. The accounts from general practitioners show that there was concern about skills relating to communication, cultural, organisational and clinical issues. The accounts from the health promotion and public health nursing staff provide an excellent insight into the ways in which mainstream programmes and services may not always be relevant to the needs of asylum seekers who require specialised and targeted care in the community. This lack of skills, including clinical skills, needs to be addressed at an organisational level within the Health Service Executive to enhance the capacity of primary healthcare delivery to the asylum-seeker/refugee communities. Also, it is problematic that frontline providers who *are *skilled and expert professionals were in a situation in which they felt unskilled and unconfident about their work. This finding is similar to previous research [19] and more recent studies [41-43] showing that this is a persisting problem which requires attention from relevant professional and educational bodies in Ireland and abroad.

The sudden opening of Accommodation Centre A also led to *practical problems*. Overcrowding in waiting rooms was problematic for frontline providers, but also local service users. This is not a positive context for integration of new communities into our society. However, that said, it was striking that the experience of asylum seekers in the study area was broadly positive. They did not report experiences of negativity or hostility and commented that, in fact, local community members were nice to them. This is surprising because other studies in Ireland have reported asylum seekers' and other migrants' experiences of racism and discrimination which is negative for mental health and wellbeing [44]. Perhaps the fact that this group of asylum seekers were fortuitous to have encountered a GP, public health nurses, a health promotion worker and social care workers who were very committed to them and their needs influenced their perceptions of friendliness in the community.

Service providers described the use of *formal and informal strategies *for managing the challenges they experienced. We have learned that a number of effective informal meetings took place before and after formal ones at which service providers from statutory and non-statutory organisations worked very hard to devise plans to ensure an effective response for the newly arrived asylum seekers. There are examples of GPs meeting and negotiating a division of labour for the care of asylum seekers. Primary care colleagues met and created informal alliances to manage the situation. NGOs liaised with each other to organise support for the asylum seekers. As a result of these negotiations and divisions of labour, some frontline staff took on new roles and responsibilities to manage the situation. They worked hard to ensure that there was not a gap in service provision, often going 'beyond the call of duty' to do so. The result was a number of interdisciplinary networks that operated effectively on the ground. It is interesting and encouraging to consider that the asylum seekers report positive experiences of general practice care and public health and health promotion activities.

However, despite some positives, it is clear that what emerged was an *ad hoc response*. Furthermore, this ad hoc response was contingent on interpersonal and inter-professional alliances and networks and good will within those. The example of one GP taking on the whole community of asylum seekers is a stark illustration of this point. It was positive in the sense that the asylum seekers had access to general practice care (and we know that they were very satisfied with that care) but it was problematic because, with almost a 'twist of fate,' their experience of general practice could have been radically different. What if the GPs refused to take any of the asylum seekers on as GMS patients? This is a real and documented scenario for asylum seekers in other parts of Ireland [45] and abroad [18,46]. This is clearly in breach of international policies by the UN and WHO emphasising the rights of asylum seekers to access health care [2,7].

### Methodological critique

We obtained a sample of 37 participants, which is large for a qualitative study. We highlight that there was very good representation across participant groups. We were particularly pleased to have participation from the asylum seekers who were residents in Accommodation Centre A because they had moved from the area at the time of the research. It was important and valuable to make appropriate efforts to include their voices in the case study.

We used semi-structured interviews to gather data from representatives of all stakeholder groups. Although these are retrospective in nature and based on people's recollections of past events, we note the concordance of accounts from different participant groups, and also the resonance of the accounts with available documentation from that time (e.g. media reports). Through these interviews, we obtained in-depth descriptions of participants' experiences and their reflections on these. The research team involved in data collection and analysis comprised a GP researcher (HOPieper) and two social scientists (AMacFarlane, PClerkin). This multidisciplinarity is known to enhance qualitative analysis and is highlighted here as a positive feature of the research process [47].

Another interesting feature of the study methodology was that one of the researchers (HOPieper) was a GP involved in the case study. This offered a unique opportunity for him to provide retrospective observational data, and also to comment on the 'credibility' of the emerging findings. We are also aware, however, that his immersion in the case might have influenced data collection, perhaps limiting what certain stakeholders would say about their experiences. Therefore, a portion of interviews (10%) were conducted by PClerkin. Also, the analysis has been shared with participants to allow them the opportunity to comment on the accuracy of our descriptions of their experiences and the credibility of our interpretations.

## Conclusion

Dispersal policies and direct provision accommodation have been linked to violation of basic human rights, poverty, and poor physical and mental health [6,10-14]. This study adds to our understanding because it focuses on the impact of these policies on the organisation and delivery of primary care and social care services. The findings highlight that organisation of services was reliant on informal divisions of labour and negotiations of professional roles and responsibilties, which were resolved in an *ad hoc *manner rather than through formal planning and co-ordination of service delivery. The findings highlight that there were few formal supports for service providers and we note that there was no formal mechanism in place to evaluate service user or service provider experiences.

To address the problems described in this research, it is recommended that there is greater inter-sectoral interaction between policy makers, service planners, service providers and community-based organisations so that the effects of immigration policy (e.g. direct provision policy) are considered and negotiated *vis a vis *other relevant policy (e.g. intercultural health policy) and healthcare practices (e.g. access to care, good informational continuity). Dialogue at an inter-sectoral level should lead to more formal planning and inter-agency collaboration on the ground between statutory and non-statutory agencies and within the health services. In addition, it is recommended that there is a renewed effort to improve intercultural training and education for primary care and social care providers so that persisting findings about professional uncertainty about working in cross-cultural consultations is addressed effectively, with benefits for health and social care providers and the asylum seekers (and other migrants) for whom they care.

## Competing interests

The authors declare that they have no competing interests.

## Authors' contributions

HOP was project researcher leading on data collection and analysis and write up. PC contributed to data collection and analysis. AMacF was Principal Investigator. She designed the study, oversaw data collection, and participated in data analysis and project write up. All authors read and approved the final manuscript.

## Pre-publication history

The pre-publication history for this paper can be accessed here:

http://www.biomedcentral.com/1471-2296/12/32/prepub
